# Stability Testing of a Wide Bone-Anchored Device after Surgery without Skin Thinning

**DOI:** 10.1155/2015/853072

**Published:** 2015-07-05

**Authors:** Malou Hultcrantz

**Affiliations:** Department of Otorhinolaryngology, Karolinska University Hospital, 171 76 Stockholm, Sweden

## Abstract

*Objective*. To longitudinally follow the osseointegration using Resonance Frequency Analysis (RFA) for different lengths of abutment on a new wide bone-anchored implant, introduced with the non-skin thinning surgical technique. *Study Design*. A single-center, prospective 1 year study following adults with bone-anchored hearing implants. *Materials and Methods*. Implantation was performed and followed for a minimum of 1 year. All patients were operated on according to the tissue preserving technique. A 4.5 mm wide fixture (Oticon Medical) with varying abutments (9 to 12 mm) was used and RFA was tested 1 week, 7 weeks, 6 months, and 12 months later. Implant Stability Quotient (ISQ), was measured from 1 to 100. Stability was compared to a group of patients (*N* = 7) implanted with another brand (Cochlear BI400) of 4.5 mm fixtures. *Results*. All 10 adults concluded the study. None of the participants lost their implant during the test period indicating a good anchoring of abutments to the wide fixture tested. Stability testing was shown to vary depending on abutment length and time after surgery and with higher values for shorter abutments and increasing values over the first period of time. One patient changed the abutment from 12 to 9 mm and another from a 9 to a 12 during the year. No severe skin problems, numbness around the implant, or cosmetic problems arose. *Conclusion*. After 1 year of follow-up, combination of a wide fixture implant and the non-skin thinning surgical technique indicates a safe procedure with good stability and no abutment losses.

## 1. Introduction

Osseointegration of titanium implants is affected by several factors such as bone quality and thickness, implant geometry, insertion torque, and the relation between burr diameter when drilling and implant diameter. Traditionally, the first Brånemark-Tjellström titanium implant for bone-anchored hearing systems had a diameter of 3.75 mm, gave good stability after a few weeks of osseointegration, and demonstrated good long-term clinical results [1–3]. A consensus from 2005 recommends that the implant can be safely loaded after 4–6 weeks, but lately, 3 weeks between surgery and loading, has been reported to be sufficient in adults with normal bone conditions [4–6]. It is known that while patients with soft bone have higher susceptibility to early implant loss, increased primary stability can potentially allow these patients to load early after the surgery [7]. In order to study the progression of implant stability after implantation, the Osstell system has been developed to incorporate Resonance Frequency Analysis (RFA) [8]. Values are influenced by firmness of the fixation, degree of osseointegration, hardness of the bone, and geometry of the implants (e.g., length and width). The RFA is transformed into Implant Stability Quotient (ISQ). The ISQ is a numerical value (1–100) where high ISQ values indicate good stability and low values a bad integration between the implant and the surrounding bone [8]. Potential benefits that arise from stability measurements include following the titanium-bone integration, deciding appropriate timing for processor loading, comparing different implant design, and foreseeing an eventual loss when stability measurements decrease.

Using the non-skin-thinning surgical technique for installation of bone-anchored hearing implants (BAHI) has shown many benefits and few negative effects. The procedure is quick, can be performed under local anesthesia, and can also be implemented in children [9–11]. It is performed as a one-step surgery which omits the skin thinning step of the classical BAHI implantation procedure, and the skin surrounding the abutment is left in its natural thickness without any scar tissue.

The first implants produced in the 1980s were 3 or 4 mm in length with a diameter of 3.75 mm and were for many years to follow installed with the skin thinning technique. A 5.5 mm abutment was always used in the thinned cutis [12, 13]. The new surgical technique without skin thinning requires an individual variation of abutment lengths suitable for the variation in skin thickness. Due to the longer leverage, the forces on the fixture can potentially be higher with a longer abutment and implants that hold a wider diameter have been introduced to the market in order to increase stability and osseointegration [14–16]. To primarily optimize and later stabilize and reduce strain on the surrounding bone, the Wide Ponto with OptiGrip geometry was launched in 2012. The implant geometry provides a large initial bone contact surface (increased by 10% compared to the previous generation) in combination with a wider implant diameter (⌀ 4.5 mm) [15]. The screw head and abutment head are designed to fit the Resonance Frequency Analysis (FRA) testing equipment.

The present study evaluates the tissue preservation surgical technique when using the new wider implant. Longitudinal testing of the RFA allowed for a review of implant stability in abutments of variable length, not earlier described.

## 2. Materials and Methods

A single-center prospective study was completed with a follow-up time of 1 year in ten consecutively operated adults, all older than 18 years of age. All participants were operated on by the same surgeon with the same osseointegrating system (Oticon Medical, Askim, Sweden). Prior to surgery, patients were tested with audiometry and were given a bone-anchored device on a soft-band to sample for 3-4 weeks.

Patients were operated on according to the tissue-preserving surgical technique without skin thinning under local anesthesia by the same surgeon. Total thickness of the skin is measured with a syringe before the skin is opened (3 cm long incision), a 3 mm or 4 mm burr is used to drill in the skull bone behind the ear to test the actual bone thickness, the opening is widened, and the predrawn fixture width and individual length of the abutment are inserted. The wide Ponto implant (⌀ 4.5 mm) with a fixture length of 4 mm was used throughout the study and abutments used were 9 or 12 mm. Thereafter a hole was punched through the entire thickness of the skin, the abutment was externalized, and the skin was closed with intracutaneous soluble sutures [9].

The patients' medical records provided information concerning clinical signs and symptoms, gender, concomitant medication, and skin diseases. Peri-implant infections, numbness around the implant, change of abutment length, abutment loss, skin overgrowth, use of hearing devices, and stability were recorded after surgery. Peri-implant infections were scored according to Holgers' scale (1–4) [17].

The Osstell system (Osstell, Gothenburg, Sweden) was used, with the help of resonance frequencies, to measure implant stability as a function of stiffness of the bone-implant interface. When testing, a rod (SmartPeg) is attached to the implanted abutment top. The probe of the Osstell instrument measures, contact-free, over a range of frequencies, by exciting the SmartPeg which starts to vibrate in the directions where highest and lowest resonance frequency occur. If there is an instable osseointegration, the vibrations will be high and give a low ISQ value, and the reverse. According to the recommendations 2 tests were always recorded at every visit, perpendicular to the peg in 2 directions 90 degrees from each other in the same plane. Each test gives a high and a low value depending on the vibrations, further used for the study analysis.

For comparison of measured stability, 7 adults implanted with another system, the Cochlear BI400, with a fixture of 4.5 mm in diameter and 4 mm length, are reported. Surgery, surgeon, and implantation technique are the same as described above, 6 with a 10 mm abutment and 1 with a 12 mm.

Appointments and checkups were arranged 1 week, 7 weeks, 6 months, and 12 months after surgery. At each time point (5 times), the RFA method was used and the 2 ISQ values were noted.

The clinical study was conducted in accordance with the ethical regulations of the Declaration of Helsinki and in adherence to Swedish law and regulations with an ethical permission (nr 2012/452-31/3).

### 2.1. Statistical Analysis

Ten was considered to be an appropriate number of operated patients followed for 1 year. Statistical analyses were carried out with Student's *t*-tests, where a *P* value less than 0.05 was considered to be statistically significant.

## 3. Results

Mean age of the patients, including 7 females and 3 males, was 54 years.  Table 1 reveals background information for all patients. Indications for surgery included mixed hearing loss (MHL) *n* = 4, single-sided deafness (SSD) *n* = 3, atresia *n* = 2, and sensorineural hearing loss (SNHL) *n* = 1. All patients were operated unilaterally, and the skin thickness varied from 6 to 12 mm. Seven patients used a 9 mm long abutment and the remaining patients received a 12 mm long abutment. In order not to have to select preoperatively for the patient which special processor they should use, the Oticon implant system is useful since this implant is compatible with both the Ponto processors and the Cochlear processors. Any brand of sound processor can therefore be utilized with the Oticon wide implants and abutments. Eight patients chose a Ponto processor (Oticon Medical, Askim, Sweden) while 2 chose a BP 100 (Cochlear, Gothenburg, Sweden). Selection of sound processors was this way based on patients' personal taste.

The most common concomitant diseases reported among the group were asthma, high blood pressure, and heart problems. Two individuals with syndromes were included (Rubenstein Taybi and Treacher Collins), as was one who suffered from Parkinson's disease.

Mean surgical time was 12.4 min, except for one male who needed minimal tissue reduction due to a skin thickness of 12 mm where surgical time was 23 min. In all patients, surgical wounds healed within 10 days after implantation. None of the patients complained of numbness at the 1-year follow-up.

A partial overgrowth was found in the male patient who had a skin thickness of 12 mm. Since there are no longer abutments available, a small revision surgery with a minor skin reduction had to be performed after 3 months in an outpatient setting.

**Figure 1:**
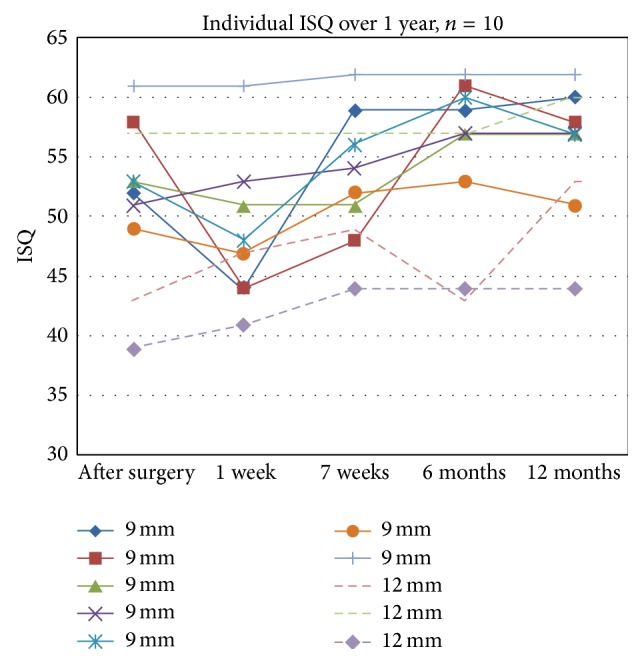
Stability test from time for surgery to the endpoint at 1 year, showing individual ISQ values for 10 patients. High ISQ values are shown.

The ISQ stability test across all 10 patients varied over time from a starting median value of 52 and 51, high and low, respectively, to 56 and 54 after 1 year. The ISQ values decreased at 1 week after surgery compared to the value at installation, which was statistically significant (*P* < 0.05), and seem to have reached a plateau around 3 months later. In the group of only 9 mm abutments (*n* = 7), the initial values were 54 and 53 (range 43 to 61) and these values increased after 1 year to 58 and 54 (range 46 to 60). In the 12 mm abutment group (*n* = 3), the values measured were 45 and 44 (range 39–57) postsurgically and 51 and 49 (range 43 to 60) after 1 year (Figure 1).

When comparing the stability test from the 7 control patients the ISQ values were for the 10 mm abutment length initially 46.5 and after 1 year 52.5 and for the 12 mm (only *n* = 1) 45 and 48, respectively.

**Figure 2:**
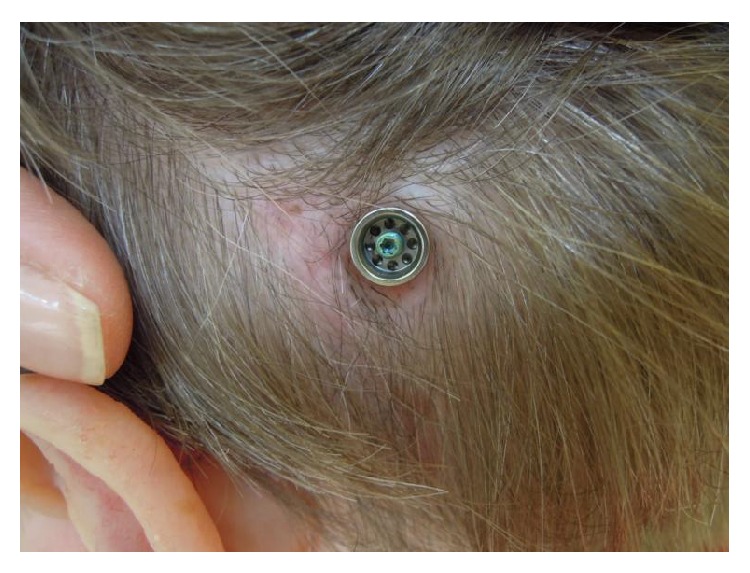
Implant with a 9 mm abutment 3 months after surgery. The abutment was recently changed from 12 to 9 mm. ISQ high and low values increased minimally from 44 and 45 to 46 and 46.

Change of abutment was performed, as an outpatient procedure in two patients. One female patient had a 9 mm abutment replaced by a 12 mm, and one male patient had a 12 mm abutment changed to a 9 mm so as to better fit the processor (Figure 2). The measured ISQ values changed minimally.

No implants were lost and no abutments were permanently removed.

One minor skin reaction was noted in one female patient 3 months after surgery, with a Holgers' scale scoring 2. This patient was treated with extra cleaning and lapisation (silvernitrate), and no further problems were experienced. None of the other patients had any skin reaction during the 1 year follow-up period.

## 4. Discussion

The current follow-up study is a 1-year prospective clinical trial designed to evaluate stability in a new wider fixture, fitted with variable length of abutments in combination with the tissue preservation surgical technique which has not been reported earlier. Patients have excellent results subjectively and objectively and almost all (nine out of ten) were wearing their processor daily after 1 year.

Prior to the current study, research suggested that the non-skin-thinning surgical technique is beneficial when implanting BAHIs in both children and adults and many of the earlier known complications disappeared or were reduced [9–11, 16]. The present study confirms and reinforces those results. The longer abutments required for this technique can increase the force transferred to the fixture with higher demand on osseointegration and stability. The manner in which stability varies between the different abutments and fixtures has not been fully reported clinically. The new wide Ponto implant showed improved stability in the laboratory, and the current study confirms that this improved stability leads to few implant losses [15].

There are now a greater number of studies reporting ISQ values in the literature, but still no consensus has been reached as to what exact level an implant is considered to be fully osseointegrated. Indications suggest that levels of ISQ around 60 demonstrate normal values for good stability, but most information gathered is reported with older, shorter abutments (5.5 or 6 mm). For comparison implantation with a 4.5 mm wide BIA300 implant (Cochlear, Gothenburg, Sweden) showed with the linear incision technique (skin thinning) and 5.5 mm abutments a ISQ of 62 after 6 months which is in the range of ISQ reported presently, but here with longer abutments and a higher level of the skin around the abutment [18]. Comparing the present wide implant stability with older more narrow ones is impossible since the SmartPeg for testing ISQ does not fit the older abutments.

Caution must be taken when comparing specific ISQ values, as ISQ values in a soft bone, although well-integrated, give lower ISQ values than an insufficiently integrated implant in hard bone [19]. The highest survival rates in implants after osseointegration are reported from stability tests after placement in the temporal region as compared to other bones [20–22]. Research has also shown that poorer bone quality, younger children, and syndromic patients have a higher extrusion rate, indicating that behavioral and biological factors must be taken into account [23–25]; however, high success rates in implantations have been reported after irradiation and also in low mineralized bone [26–28].

Basic studies in the laboratory indicate ISQ values with the same implant but different abutment lengths increasing from 46 (12 mm abutment) to 54 (9 mm) and 62 (6 mm), respectively, giving an estimation of stability loss of 3 to 4 ISQ for each mm of longer abutment [15]. Higher values in a shorter abutment can also be confirmed in the present study and also when compared to the control group. In the 9 mm abutment group the difference of ISQs over the year increased totally with 4 (ISQ high) and with 6 in the 12 mm group (not statistically significant). Among the controls the 10 mm abutment group increased with 6 and the 12 mm group with 3 ISQ units during the one year tested. These values indicate small, but increasing values over time and also comparable results for long abutments. Increasing ISQ values during the first part of the illustrated year and a difference between abutment lengths are noted but are not remarkable and the number of patients in each group is low. Exact values cannot be compared to other studies, due to differences in abutment length, surgical technique, bone quality, and position of the fixture, but when compared with dental studies, using different abutment lengths, increasing values are confirmed over time [14]. Recent studies of early loading with a 5.5 mm abutment showed that all included patients had values over 60, 1 week after surgery, with increasing values over time, demonstrating almost immediate implant stability [6, 29].

When considering the fixtures and their different lengths, it has been shown in a group of pediatric implantations that there was no favor reported for a 4 mm fixture, 3.75 mm in diameter, over a 3 mm fixture with the same diameter in number of osseointegration failure rates [16]. When the fixtures were placed transcalvarian, the 3 mm fixtures were even more stable. Total osseointegration failure rates were 21% in children as compared to 0% among adults which are not surprising when considering the softness of the skull bone in developing children. However, good stability and indications for early loading are given to be ISQ > 60 in children [30]. New information of implanted children with longer abutments demonstrates that a low ISQ value of 30 could indicate a value where an implant loss can be considered [24].

Individual patient factors possibly play a larger role in the failures than the surgical implantation and fixture length. Presently, only adult patient was included and the wide fixture seems to be pertinent to hold both abutment lengths tested based on stability tests, due to the fact that no implant was lost. The implant design and the surgical technique may account for the high survival rates. New studies will be needed to confirm the statistical analysis, since the number of patients included is low and further follow-up of patients will be reported.

## 5. Conclusion

The present prospective, one year follow-up study performing a BAHI surgical technique without skin thinning in ten consecutively operated patients, reveals that the non-skin-thinning technique in combination with a wide implant (4.5 mm in diameter) provides few complications. The absence of implant losses indicates a safe procedure for the patients. The ISQ values indicate small differences between the wide fixture with either 9 or 12 mm abutments all with good stability (ISQ 54 and 57, resp.) 1 year after implantation.

## Figures and Tables

**Table 1:** Demographics from 10 patients implanted with a wide implant, during a 1-year period.

Age	Indication	Abutm. loss	Peri-impl. inf.	Holgers scale	Numbness	Side	Skin thick-ness mm	Abutm. length mm	Abutm. change	ISQ start	ISQ end
58	SSD	—	—		—	L	6	9		52,49	60,59
46	Atresia	—	—		—	L	10	12		43,43	53,51
87	EO, SNHL	—	—		—	L	6	9		58,57	58,57
65	SSD	—	—		—	R	8	9		57,55	60,60
39^*^	COM	—	—		—	R	12	12		53,51	57,57
45	SSD	—	Yes	2	—	R	6	9		51,49	57,57
61	COM	—	—		—	R	7	9		53,54	57,56
59	COM	—	—		—	L	7	9		49,46	51,30
35	COM	—	—		—	L	6	9	9 to 12	61,58	61,62^**^
46	Atresia	—	—		—	L	9	12	12 to 9	39,39	46,46^**^

Total										52,51	56,54^***^

^*^Patient with 12 mm thick skin where a minor skin thinning was performed.

^**^Patient started with 1 abutment length and ended with another.

^***^ *n* = 8, 2 patients changed the abutment length during the study.
